# Progesterone administration does not acutely alter LH pulse secretion in the mid‐follicular phase in women

**DOI:** 10.14814/phy2.13680

**Published:** 2018-04-25

**Authors:** Su Hee Kim, Christine M. Burt Solorzano, Christopher R. McCartney

**Affiliations:** ^1^ Division of Endocrinology and Metabolism Department of Medicine University of Virginia School of Medicine Charlottesville Virginia; ^2^ Center for Research in Reproduction University of Virginia School of Medicine Charlottesville Virginia; ^3^ Division of Endocrinology and Metabolism Department of Pediatrics University of Virginia School of Medicine Charlottesville Virginia

**Keywords:** Gonadotropin‐releasing hormone, luteinizing hormone, negative feedback, progesterone

## Abstract

It remains unclear how rapidly progesterone suppresses luteinizing hormone (LH) pulse frequency in women. Previous studies suggested that progesterone markedly increases LH pulse amplitude but does not slow LH pulse frequency within 10 h in estradiol‐pretreated women studied during the late follicular phase. However, this experimental paradigm may be a model of preovulatory physiology, and progesterone may have different effects at other times of the cycle. We studied regularly cycling, nonobese women without hyperandrogenism to assess the acute effect of progesterone during the midfollicular phase and in the absence of estradiol pretreatment. The study involved two admissions in separate cycles (cycle days 5–9). For each admission, either oral micronized progesterone (100 mg) or placebo was administered at 0900 h in a randomized, double‐blind fashion. Frequent blood sampling was performed between 0900 and 1900 h to define 10‐h LH pulsatility. Treatment crossover (placebo exchanged for progesterone and vice versa) occurred in a subsequent cycle. After an interim futility analysis, the study was halted after 7 women completed study. Mean progesterone concentrations after placebo and progesterone administration were 0.5 ± 0.1 (mean ± SD) and 6.7 ± 1.6 ng/mL, respectively. Compared to placebo, progesterone was not associated with a significant difference in 10‐h LH pulse frequency (0.79 ± 0.35 vs. 0.77 ± 0.28 pulses/h, *P* = 1.0) or amplitude (3.6 ± 2.8 vs. 4.3 ± 2.8 IU/L, *P* = 0.30). This study suggests that LH pulse frequency is not rapidly influenced by progesterone administration during the midfollicular phase.

## Introduction

Gonadotropin‐releasing hormone (GnRH) stimulates luteinizing hormone (LH) and follicle‐stimulating hormone (FSH) synthesis and secretion. High GnRH pulse frequencies favor LH secretion and low GnRH pulse frequencies favor FSH secretion (Wildt et al. 1981; Gross et al. 1987; Spratt et al. 1987), and an ability to modulate GnRH pulse frequency appears to be important for the normal cyclic patterns of LH and FSH secretion (Cook et al. 1991). Progesterone is the primary modulator of GnRH pulse frequency slowing in women. For example, LH (and by inference GnRH) pulse frequency slows as progesterone increases in the luteal phase (Filicori et al. 1984, 1986), and administration of progesterone for 8 days slows LH pulse frequency in women studied during the follicular phase (Soules et al. 1984). Although progesterone suppresses GnRH pulse frequency within 6 h in sexually mature ewes and cows (Bergfeld et al. 1996; Skinner et al. 1998), the rapidity with which progesterone suppresses LH pulse frequency in women is uncertain.

We have previously studied the acute effect of progesterone administration in normal women assessed during late follicular phase (cycle days 7–11) after estradiol‐pretreatment for 3 days (McCartney et al. 2007; Hutchens et al. 2016). In these studies, a single 100 mg oral dose of progesterone was administered at either 1800 h (McCartney et al. 2007) or 0600 h (Hutchens et al. 2016). In both studies, LH pulse frequency was not suppressed within 12 h, but mean LH and LH pulse amplitude increased markedly. However, we considered the possibility that this experimental paradigm – assessment during the late follicular phase after estradiol pretreatment – may be a model of preovulatory physiology, and that progesterone may have different effects on LH pulse frequency at other times of the cycle. As such, we performed a study to test the hypothesis that progesterone reduces LH pulse frequency in women assessed during the midfollicular phase in the absence of exogenous estradiol pretreatment.

## Materials and Methods

The Institutional Review Board at the University of Virginia (UVA) approved all study procedures, which were in accordance with the ethical standards of the Helsinki Declaration of 1975, as revised in 2008. The study was registered with ClinicalTrials.gov (identifier NCT 01602679).

### Subjects

Seven healthy, nonobese women with regular menstrual cycles and no evidence of hyperandrogenism completed the study and were included in the analysis (Table 1). None of the subjects reported excessive exercise or recent weight loss. No subject had taken medications known to affect the reproductive axis for 90 days prior to or during the study.

**Table 1:** Subject characteristics

	Mean	SD	Median	Range
Age (years)	20.2	1.9	20	18–23
Cycle length (days)	29.0	1.4	29	27–31
BMI (kg/m^2^)	23.1	2.4	23.4	18.7–26.8
Body fat percentage (%)	27.6	6.8	29.9	16.9–35.3
Waist‐to‐hip ratio	0.75	0.05	0.74	0.70–0.82
Total testosterone (ng/dL)^a^	16.7	7.3	17.6	5.3–26.4
SHBG (nmol/L)^a^	53	29.7	41.4	25.8–109.5
Calculated‐free testosterone (pg/mL)^a^	2.4	1.5	1.9	1.1–5.4
Estradiol (pg/mL)^a^	25.1	9.6	20.6	15.9–42.3
Fasting insulin (*μ*IU/mL)	5.1	3.3	4.1	2.0–9.6
Fasting glucose (mg/dL)	84	6.6	84	75–94

The number of subjects is 7 for all variables. To convert conventional to SI units: total testosterone (ng/dL) × 3.467 (nmol/L); SHBG (*μ*g/mL) × 8.896 (nmol/L); free testosterone (pg/mL) × 3.467 (pmol/L); estradiol (pg/mL) × 3.671 (pmol/L); insulin (*μ*IU/mL) × 7.175 (pmol/L); glucose (mg/dL) × 0.0555 (mmol/L).

BMI, body mass index; SHBG, sex hormone‐binding globulin; SD, standard deviation.

a Reported values were obtained from the placebo admission.

### Study procedures

After full, written informed consent was obtained, subjects underwent a detailed screening history and physical examination, and laboratory testing to screen for hormonal and health‐related abnormalities, as previously described (McCartney et al. 2007; Hutchens et al. 2016). BOD POD^®^ was used to assess percent body fat. Waist and hip circumference were also measured.

The study followed a randomized, placebo‐controlled, double‐blinded, crossover design with assessment of the acute effects of progesterone and placebo (individually) on pulsatile LH secretion. All admissions occurred in the midfollicular phase (cycle days 5–9 inclusive). Red blood cell counts and *β*‐hCG were checked 1–3 days before each admission to exclude anemia and pregnancy, respectively. Each subject underwent two separate admissions for frequent blood sampling from 0900 to 1900 h in the UVA Clinical Research Unit (CRU): LH every 10 min; progesterone every 30 min for 4 h, then every 2 h; FSH, estradiol, testosterone every 2 h. SHBG was measured once at 0900 h. Subjects were randomized to receive either oral micronized progesterone (100 mg) or placebo at 0900 h (immediately before the first blood draw) during the first admission. Investigators, research staff, and subjects were blind to treatment allocation. Subjects were not allowed to sleep during the admission and were asked to eat only the meals provided by our CRU staff during the admissions. Subjects were discharged after the final blood draw at 1900 h.

A second CRU admission occurred during a subsequent menstrual cycle. This admission was identical to the first except that placebo administration was exchanged for progesterone administration or vice versa in accordance with the crossover design.

### Hormonal measurements

All hormone assays were performed by the Ligand Assay and Analysis Core of the Center for Research in Reproduction as previously described (Hutchens et al. 2016). Briefly, LH was measured by chemiluminescence (sensitivity 0.1 IU/L; intraassay coefficient of variation [CV] 3.3%; interassay CVs 5.8%; Siemens Healthcare Diagnostics, Los Angeles, CA). FSH and progesterone were measured by chemiluminescence, while total testosterone and estradiol were measured by radioimmunoassay; sensitivities, intra‐ and interassay CVs were as described previously (Hutchens et al. 2016). All samples from an individual woman were analyzed in duplicate in the same assay for each hormone. Measured hormone concentrations below assay sensitivity were assigned the value of the assay's sensitivity. To convert from conventional to Systeme International (SI) units: progesterone × 3.18 (nmol/L); total testosterone × 3.47 (pmol/L); estradiol × 3.671 (pmol/L).

### Data analysis

Assessments of pulsatile LH secretion were performed by a single investigator (CRM) while blinded to treatment condition. As previously described (Hutchens et al. 2016), we employed a computerized data reduction protocol (StdCurve) to establish a variance model for each LH concentration time series; this procedure provided statistically accurate estimates of experimental measurement error. Thereafter, pulsatile LH secretion was characterized using AutoDecon, a fully automated multi‐parameter deconvolution program (Johnson et al. 2008). To limit false positives, we excluded AutoDecon‐identified pulses that did not demonstrate either (1) at least two peak values that were at least 10% higher than the preceding nadir, or (2) at least one peak value that was at least 20% higher than the preceding nadir. The temporal locations of LH pulses were used to calculate average interpulse interval (IPI) over the sampling period as previously described (McCartney et al. 2007; Kim et al. 2018). Then, LH pulse frequency (pulses per hour) was calculated as 60 divided by the average IPI. We also calculated average LH pulse amplitude and average LH pulse mass – an AutoDecon‐derived estimate of the amount of LH released by the pituitary during each secretory episode.

### Statistical analysis

The primary endpoint for this study was the change in LH pulse frequency attributable to progesterone, defined as the 10‐h LH pulse frequency under the progesterone condition minus the 10‐h LH pulse frequency under the placebo condition. Our a priori hypothesis was that 10‐h LH pulse frequency after progesterone administration would be lower than LH pulse frequency after placebo administration. We estimated that a sample size of 12 would provide 80–90% statistical power to detect a 16.7‐min or greater difference in average LH interpulse interval (progesterone vs. placebo), assuming a within‐subject standard deviation of 20.6 min. However, given negative results in each of our prior two studies (McCartney et al. 2007; Hutchens et al. 2016), we performed an interim assessment after seven women had completed study. This interim assessment suggested no pulse frequency differences between progesterone and placebo conditions (*P* = 1.0 by Wilcoxon signed rank test). To assess the potential utility versus futility of full study completion (i.e., the study of 5 additional subjects to reach our planned *n* = 12), we performed a Wilcoxon signed‐rank test on the existing 7 observations plus 5 fabricated observations, with each fabricated observation stipulated to demonstrate a pulse frequency reduction with progesterone that exceeded the largest reduction observed in the existing 7 study subjects. Since this Wilcoxon signed‐rank test only yielded a *P*‐value of 0.0522, we concluded that study continuation to our initially targeted sample size was futile, and we halted the study.

As preplanned secondary statistical analyses, differences in average 10‐h LH pulse amplitude, LH pulse mass, mean LH, and mean FSH between the placebo and progesterone admissions were analyzed, using Wilcoxon rank sum tests. Differences in mean progesterone, estradiol, and total testosterone concentrations were also analyzed using Wilcoxon signed‐rank tests.

We performed post hoc analyses to assess whether progesterone affected LH pulse parameters – especially LH pulse frequency – across the 10‐h period of observation. Firstly, we used Wilcoxon signed‐rank tests to assess differences in LH pulse frequency, LH pulse amplitude, LH pulse mass between progesterone and placebo conditions in two separate 5‐h time blocks: 0900–1400 and 1400–1900 h. We also determined whether temporally‐specific estimates of LH pulse frequency (“instantaneous pulse frequency”) differentially changed across the 10‐h observation period under the progesterone and placebo conditions. Specifically, for each fully‐defined IPI, instantaneous pulse frequency was calculated as 60 divided by the IPI and assigned a temporal location at the midpoint of each IPI. For example, if three pulses occurred at 1000, 1200, and 1600 h – rendering two IPIs of 120 and 240 min – associated instantaneous pulse frequencies would be 0.5 and 0.25 pulses/h located at 1100 and 1400 h, respectively. For each subject and each treatment condition (progesterone vs. placebo), we used simple linear regression to assess whether instantaneous LH pulse frequency tended to decrease or increase from 0900 to 1900 h. We then performed a Wilcoxon signed‐rank test to assess for treatment‐related differences in regression line slopes. As final post hoc analyses, we used Spearman rank correlation to assess whether the change in 10‐h LH pulse frequency potentially attributable to progesterone (i.e., LH pulse frequency during the progesterone admission minus LH pulse frequency during the placebo admission) was related to either (1) testosterone concentration during the placebo admission or (2) estradiol concentrations.

SAS version 9.4 (SAS Institute Inc., Cary, NC) was used for all statistical analyses. We performed nonparametric statistical tests, which are based on ranks of observations and require no assumptions about underlying data distribution. A two‐sided *P* ≤ 0.05 decision rule was used as the null hypothesis rejection criterion for all statistical tests. Data are presented as mean ± SD unless indicated otherwise. Subject‐level data are provided in Supplemental Materials.

## Results

Progesterone and placebo admissions occurred on cycle days 5.9 ± 1.1 and 6.1 ± 1.3, respectively. As determined by simple randomization, progesterone was given during the first admission for 4 subjects, and placebo was given during the first admission for 3 subjects.

### Sex steroids

**Figure 1:**
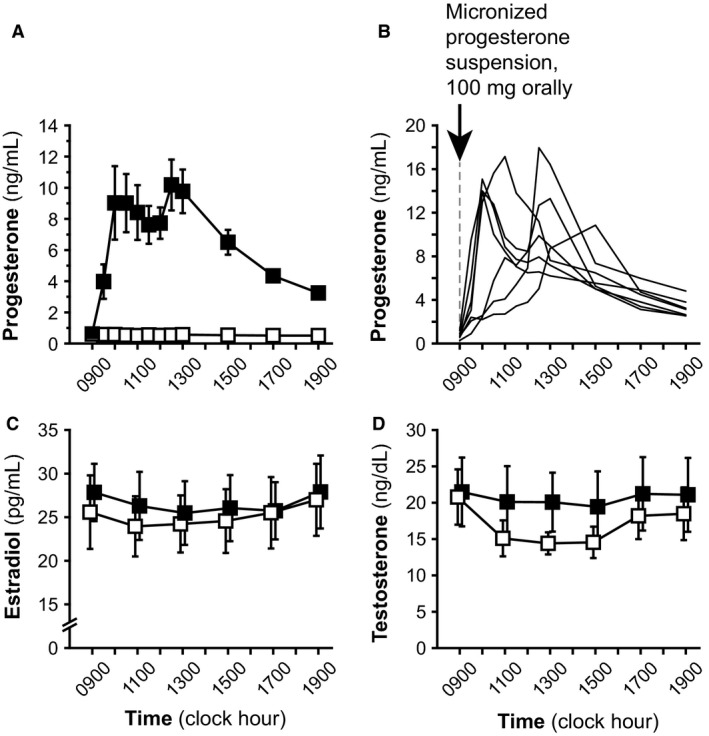
Progesterone (panels A, B), estradiol (panel C), and total testosterone (panel D) concentrations. Progesterone and placebo admissions are denoted by solid and open data points, respectively. In panels (A), (C), and (D), data are shown as mean ± standard error of the mean. Panel (B) shows each individual's progesterone levels after exogenous progesterone ingestion at 0900 h. Conversion from metric to Systeme International (SI) units: progesterone × 3.18 (nmol/L); estradiol × 3.67 (pmol/L); total testosterone × 0.0347 (nmol/L).

Summary data for progesterone, estradiol, and total testosterone concentrations are presented in Table 2, and sex steroid values during each admission are graphically represented in Figure 1. As intended, mean progesterone concentrations were higher during progesterone admissions compared to placebo admissions (6.7 ± 1.5 vs. 0.5 ± 0.1 ng/mL, respectively; *P* = 0.0156). Mean estradiol and total testosterone concentrations were similar between admissions (*P* = 0.5781 and 0.5625, respectively).

**Table 2:** Summary statistics, sex steroid concentrations. Summary statistics are partitioned by treatment condition (progesterone vs. placebo)

	Treatment condition	Mean	SD	Median	Range
Progesterone (ng/mL)	Placebo	0.5	0.1	0.5	0.3–0.7
	Progesterone	6.7	1.5	4.5	4.2–9.1
Estradiol (pg/mL)	Placebo	25.1	9.6	20.6	15.9–42.3
	Progesterone	26.5	9.2	24.2	13.6–41.0
Testosterone (ng/dL)	Placebo	16.9	6.8	17.6	6.8–26.4
	Progesterone	20.6	12.4	17.6	8.4–41.2

The number of subjects is 7 for all variables. To convert metric units to SI units: progesterone × 3.18 (nmol/L); estradiol × 3.67 (pmol/L); total testosterone × 0.0347 (nmol/L).

### LH pulse frequency

**Figure 2:**
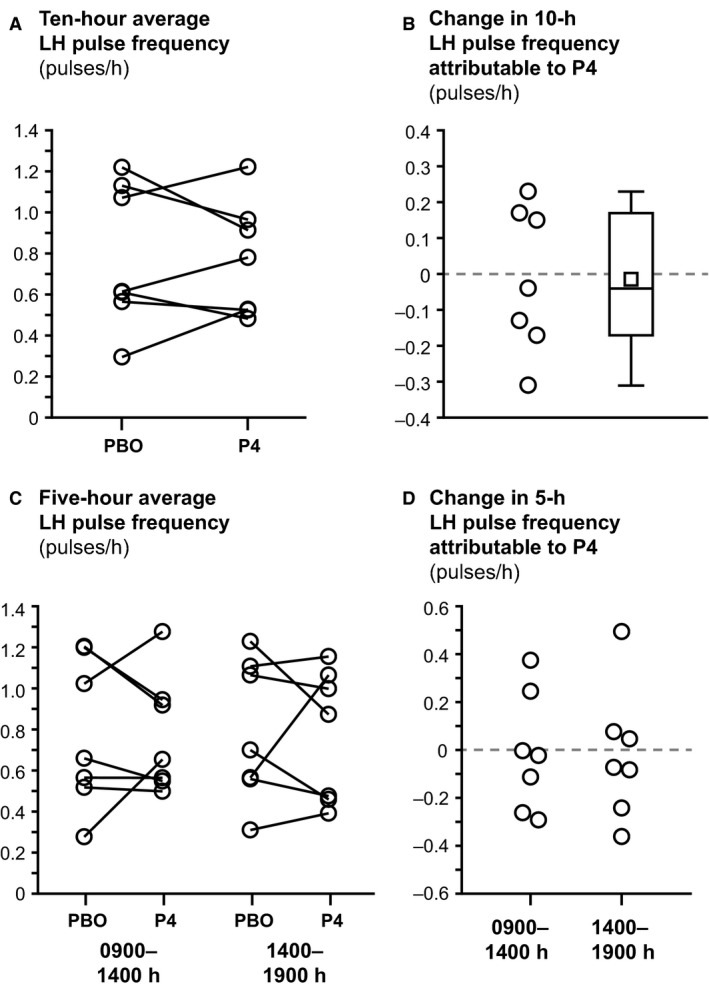
LH pulse frequency. Panel (A) shows each subject's 10‐h average LH pulse frequency under the placebo (PBO) and progesterone (P4) conditions. In panel (B), the open circles represent the change in 10‐h LH pulse frequency attributable to progesterone (i.e., 10‐h LH pulse frequency under the P4 condition minus the 10‐h LH pulse frequency under the PBO condition). These data are also summarized using box‐and whisker plots (median [line inside the box], 25th and 75th percentiles [bottom and top of box], mean [open square], minimum and maximum [bottom and top whiskers]). Panel (C) shows each subject's 5‐h average LH pulse frequency under placebo and progesterone conditions in two different time blocks, 0900–1400 h (left column) and 1400–1900 h (right column). In panels (A) and (C), each subject's data are represented by connected open circles. In panel (D), open circles represent the change in 5‐h LH pulse frequency that is attributable to progesterone in the respective 5‐h time blocks.

Summary data for LH pulse frequency are shown in Table 3 and represented graphically in Figure 2. There was no significant difference in 10‐h LH pulse frequency between progesterone and placebo admissions (0.77 ± 0.28 and 0.79 ± 0.35 pulses/h, respectively; *P* = 1.0; Fig. 2A and B). No differences were observed for either 5‐h time block (*P* > 0.6 for both; Fig. 2C and D).

**Table 3:** Summary statistics, gonadotropin characteristics. Summary statistics are partitioned by treatment condition (progesterone vs. placebo). The number of subjects is 7 for all variables

	Treatment condition	Mean	SD	Median	Range
LH pulse frequency (pulses/h)	Placebo	0.79	0.35	0.61	0.30–1.22
	Progesterone	0.77	0.28	0.78	0.48–1.22
Mean LH (IU/L)	Placebo	4.6	2.3	4.2	2.5–9.5
	Progesterone	5.8	2.2	5.2	2.9–9.1
LH pulse amplitude (IU/L)	Placebo	3.6	2.8	2.7	1.1–9.5
	Progesterone	4.3	2.8	3.0	2.2–8.5
LH pulse mass (IU/L)	Placebo	4.8	3.0	3.9	1.8–11.0
	Progesterone	5.9	3.7	4.1	3.1–11.5
Mean FSH (IU/L)	Placebo	5.1	0.9	4.7	4.4–6.5
	Progesterone	5.4	1.2	5.3	3.9–6.8

**Figure 3:**
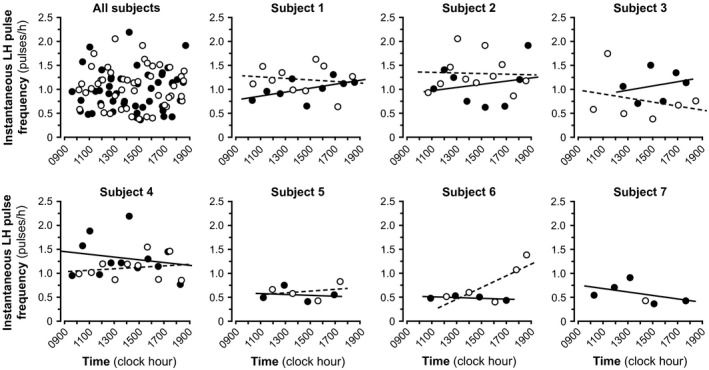
Instantaneous LH pulse frequency as a function of time. Graphs including all subjects (top left) and graphs for individual subjects (all others) are shown. Data points indicate an instantaneous LH pulse frequency at a given time under progesterone (closed circles) and placebo (open circles) conditions. Linear regression lines are drawn using solid lines for progesterone conditions and dotted lines for placebo conditions. Note that the slope of the progesterone regression line was more positive than the placebo regression line in subjects 1–3 and more negative in subjects 4–6, suggesting no consistent difference between treatment conditions. A regression line could not be calculated under the placebo condition for subject 7 because only one fully defined interpulse interval was observed during that admission.

The temporal progression of instantaneous LH pulse frequency over the sampling period did not differ between progesterone and placebo conditions: slopes were −0.0012 ± 0.0358 (0.0094) and −0.0238 ± 0.0759 (−0.0010) (mean ± SD [median]) for the progesterone and placebo admissions, respectively (*P* = 1.0, Fig. 3).

**Figure 4:**
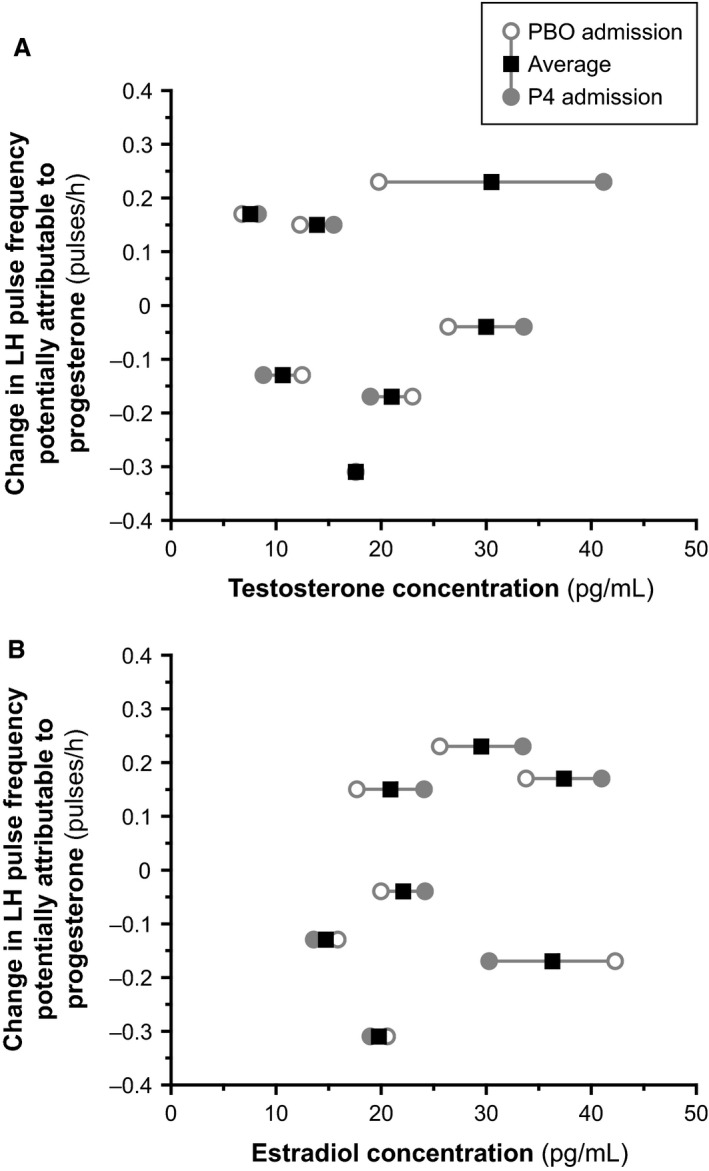
Change in LH pulse frequency potentially attributable to progesterone (i.e., pulse frequency during the progesterone [P4] admission minus pulse frequency during the placebo [PBO] admission) as a function of testosterone concentrations (panel [A]) and estradiol concentrations (panel [B]). Similar results were obtained when using calculated‐free testosterone in lieu of total testosterone.

Spearman rank correlation did not disclose a significant relationship between total or free testosterone concentrations during the placebo admission and the change in 10‐h LH pulse frequency potentially attributable to progesterone (*P* > 0.1 for both; Fig. 4A). Similarly, the change in 10‐h LH pulse frequency potentially attributable to progesterone was not related to estradiol concentrations (*P* > 0.5; Fig. 4B).

### LH pulse amplitude, LH pulse mass, and mean gonadotropin concentrations

**Figure 5:**
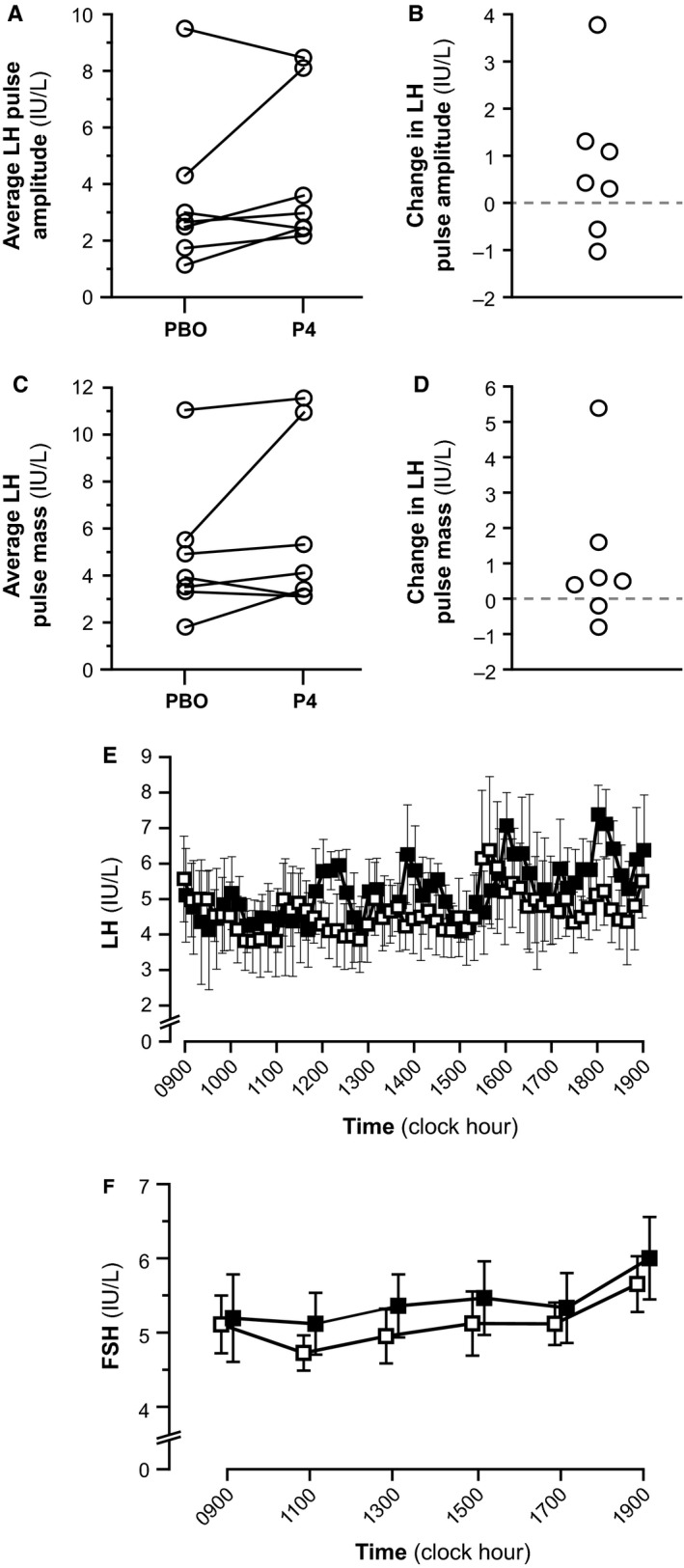
LH pulse amplitude, LH pulse mass, mean LH, and mean FSH. Panels (A) and (C) illustrate individual average LH pulse amplitude and LH pulse mass, respectively, between the placebo (PBO) and progesterone (P4) conditions. In panels (A) and (C), each subject's data is represented by connected open circles. Panels (B) and (D) show the change in LH pulse amplitude and LH pulse mass, respectively, that is attributable to progesterone. Panels (E) and (F) show mean LH and FSH, respectively, at each sampling time under placebo (open squares) and progesterone (solid squares) conditions. Data in panels (E) and (F) are represented as mean ± standard error of the mean.

Summary data for LH pulse amplitude, LH pulse mass, mean LH, and mean FSH are shown in Table 3 and represented graphically in Figure 5. The 10‐h average LH pulse amplitude was similar between progesterone and placebo admissions (4.3 ± 2.8 and 3.6 ± 1.8 IU/L; *P* = 0.2969). The 10‐h average LH pulse mass was also similar between progesterone and placebo admissions (5.9 ± 3.7 and 4.8 ± 3.0, respectively; *P* = 0.2188). No significant differences in LH pulse amplitude or mass were observed for either 5‐h time block.

Mean LH was similar under both progesterone and placebo admissions (5.76 ± 2.21 and 4.64 ± 2.69, respectively; *P* = 0.1250), as was mean FSH (5.42 ± 1.19 and 5.12 ± 0.85, respectively; *P* = 0.6875).

## Discussion

This study suggests that in nonobese, regularly cycling women without hyperandrogenism, progesterone administration during the midfollicular phase does not suppress daytime LH pulse frequency within 10 h. These findings are in keeping with previously reported data in normal adult women pretreated with estradiol and studied in the late follicular phase (McCartney et al. 2007; Hutchens et al. 2016).

**Figure 6:**
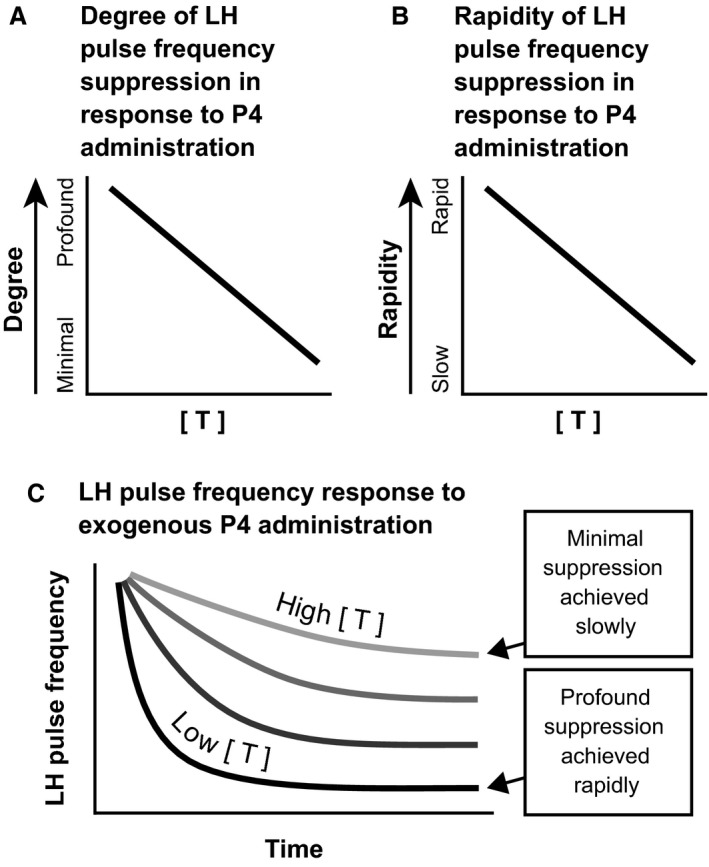
Working model regarding how the progesterone dose‐LH pulse frequency–response relationship – in terms of degree of suppression (panel [A]) and rapidity of suppression (panel [B]) – may interact with testosterone concentrations ([T]) in human females. These concepts are combined in panel (C).

The rapidity with which progesterone suppresses GnRH pulse frequency in human females remains unclear. We previously presented data suggesting that exogenous progesterone profoundly suppresses waking LH pulse frequency within 3–7 h in early pubertal girls (Collins et al. 2012), and a more recent study suggested that exogenous progesterone suppresses waking LH pulse frequency by 26% within 12–16 h in late pubertal girls during the late follicular phase (cycle day 6–11) (Kim et al. 2018). However, taken together with our previous studies (McCartney et al. 2007; Hutchens et al. 2016), the current study suggests that exogenous progesterone does not demonstrably inhibit LH pulse frequency within 10–14 h in normally cycling adult women studied during the follicular phase. The reasons for such discrepancies remain unclear. Of potential interest in this regard, androgens antagonize progesterone negative feedback (Pastor et al. 1998; Eagleson et al. 2000; Pielecka et al. 2006), and such antagonism may relate both to the *degree* of suppression – as previously described (Pastor et al. 1998; Eagleson et al. 2000) – and the *rapidity* of suppression. Thus, we hypothesize that progesterone negative feedback occurs more rapidly when androgen concentrations are very low (as in early puberty), but more slowly when androgen concentrations are higher (as in late puberty and adulthood) (Fig. 6). Such differences could also relate to the duration of exposure to higher physiologic androgen concentrations, or they could reflect other developmental changes altogether. Although we did not observe a relationship between testosterone concentration (placebo admission) and the change in LH pulse frequency potentially attributable to progesterone in this study, we believe that additional study is required before making firm conclusions.

Mean estradiol concentrations were 25–27 pg/mL in this study. Since progesterone action at the hypothalamus appears to require the permissive presence of estradiol (Karsch et al. 1973; Nippoldt et al. 1989), it remains possible that progesterone had no acute effect in our study because estradiol levels were insufficient to induce and/or maintain adequate hypothalamic progesterone receptors. However, the amount of estradiol required for progesterone action in women remains unclear, and we recently published a randomized, placebo‐controlled, crossover study suggesting that progesterone reduces waking LH pulse frequency in postmenarcheal adolescent girls studied in the mid‐ to late‐follicular phase (cycle days 6–11), when mean estradiol levels were 27–28 pg/mL (Kim et al. 2018). We also note that in the current study, the change in LH pulse frequency potentially attributable to progesterone did not appear to be related to estradiol concentrations (Fig. 4B).

While point estimates suggested that LH pulse amplitude, LH pulse mass, and mean LH following progesterone administration were approximately 20–25% higher compared to the placebo condition, these differences were not statistically significant. These results are in marked contrast to our most recent study of adult women assessed in the late follicular phase after estradiol pretreatment; in that study, progesterone administration was associated with a 2.5‐fold acute increase in daytime mean LH and a 2.9‐fold increase in daytime LH pulse amplitude (Hutchens et al. 2016). Our current results also contrast with other previous studies demonstrating acute positive feedback effects of progesterone on gonadotropin release when in the setting of estradiol pretreatment (Chang and Jaffe 1978; Liu and Yen 1983; Nippoldt et al. 1987). Taken together, these findings are consistent with the notion that estradiol priming is required for progesterone augmentation of gonadotropin release from pituitary gonadotropes. However, in the aforementioned study in late pubertal girls, who were studied during late follicular phase without estradiol pretreatment (Kim et al. 2018), progesterone was associated with an approximately twofold increase in LH pulse mass. Subjects in that study had estradiol levels similar to the levels observed in this study; and it is possible that the threshold estradiol level necessary for progesterone‐related augmentation of gonadotropin release may be different depending on the developmental maturation stage. Alternatively, there may be other cycle phase relevant factors that impact positive feedback effect of progesterone on gonadotropin release (Taylor et al. 1995).

In conclusion, these data suggest that in regularly cycling, nonobese women without hyperandrogenism studied during the midfollicular phase, a single dose of exogenous progesterone does not alter LH pulse frequency, LH pulse amplitude, or LH pulse mass within 10 h.

## Conflicts of Interest

The authors have no conflicts of interest to disclose.

## Data Accessibility
